# Protective Effects of *Chlorella vulgaris* Supplemented Diet on Antibacterial Activity and Immune Responses in Rohu Fingerlings, *Labeo rohita* (Hamilton), Subjected to *Aeromonas hydrophila* Infection

**DOI:** 10.3390/life13041028

**Published:** 2023-04-16

**Authors:** Jyotirmayee Pradhan, Swagatika Sahu, Basanta Kumar Das

**Affiliations:** 1 Department of Zoology, KKS Women’s College, Balasore 756003, Odisha, India; 2 Krishi Vigyan Kendra, Balasore 756026, Odisha, India; 3 ICAR-Central Inland Fisheries Research Institute, Barrackpore 700120, West Bengal, India

**Keywords:** *Aeromonas hydrophila*, bactericidal, *Chlorella vulgaris*, *Labeo rohita*, lysozyme

## Abstract

The current study focuses on the antibacterial activity and potential efficiency of dietary supplements of *Chlorella vulgaris* on the immune response, improved growth performance, and disease resistance of *Labeo rohita* fingerlings against *Aeromonas hydrophila* infection. Crude ethanolic extract of *Chlorella* and partially purified fractions of the extract were tested against two selected fish pathogens using the disc diffusion method. A total number of 360 rohu fingerlings (25 ± 2 g) were allocated to 4 treatments for 90 days. They were fed with an experimental diet containing *Chlorella* powder (0, 0.1, 0.5, and 1.0 g Kg^−1^ of a basic diet). To evaluate the non-specific immunity parameters including serum bactericidal, lysozyme activity, superoxide anion production, and biochemical and haematological indices, the fish were sampled at day 30, 60, 90, and after bacterial challenge. Mortalities of the fish were observed over 10 days post challenge with *A. hydrophila*. The protein and globulin levels of the treatment group were significantly higher after being treated with *Chlorella* than those of the control group. The total blood-cell count and haemoglobin content were also increased in the algal-diet-treated group. Among all the experimental diets, the 0.5 g Kg^−1^ *Chlorella* fed group of fish showed significantly (*p* < 0.05) increased serum bactericidal activity and superoxide anion production when compared with the control group on day 90. Maximum lysozyme activity (750.00 ± 3.27) was noticed in the 1.0 g Kg^−1^ diet fed group on day 30. The *Chlorella* treated group exhibited a better growth performance of the fish. The maximum survivability (86.5%) was recorded in the 1.0 g Kg^−1^ diet fed group at the end of the 10-day fish exposure to *A. hydrophila*. These results suggest that the optimum dietary *Chlorella* supplementation could be 0.5–1.0 g Kg^−1^ of the diet, which stimulates immunity and protects *L. rohita* from *A. hydrophila* infection.

## 1. Introduction

Fisheries play a significant socioeconomic role worldwide and make critical contributions to developments in employment, food security, and nutrition for the poor (FAO 2010). Environmental factors influence the physiological condition of cultured fish. Disease is one of the key obstructions to aquaculture, and it restricts economic and socio-economic development in different countries of the world including India [1]. However, bacterial pathogens are the most economically significant etiological agents and are frequently observed in epizootic outbreaks [2]. *Aeromonas hydrophila* is a Gram-negative motile bacterium naturally present in the aquatic environment and occasionally causes disease in cultured fish. Its pathogenicity is usually associated with physical trauma and stress, or it facilitates other infections [3]. Therefore, farmers have to be vigilant in fish husbandry techniques. Without environmental conflict, fish farms benefit from cultured fish, with better feed efficiency, better growth performance, and resistance to infectious diseases [4]. Successful aquaculture depends on proper nutrition, and the quality of a balanced diet is maintained using functional feed additives. The best feed additives are non-nutritious and include probiotics, antibiotics, antioxidants, and immunostimulants. However, considerable attention to the efficacy of functional feed additives is essential to improve feed quality, feed efficiency, and the health performance of fish [5]. Investigations should focus on an alternative approach to disease prevention through an immunostimulatory diet and feeding practices [6].

Research on feed formulation and diet optimization to enhance fish production with disease resistance is still in its infancy. The algal-based aqua feed has a beneficial effect on the physiological activity of fish, including improved muscle quality, increased digestibility, and improved resistance to disease, starvation tolerance, and growth performance [7,8].

To maintain the production and cost of fishmeal, currently, microalga is an alternative and environmentally sustainable source of feed ingredients. Among the photosynthetic organisms, microalgae have the highest areal biomass productivity and thus have a high commercial value. Microalgae biomass is rich in potential feed ingredients, containing unique bioactive compounds. *Chlorella vulgaris* a unicellular eukaryotic freshwater microalga widely distributed in nature. It can survive by photoautotrophy as well as heterotrophy, so it is easily cultured in different laboratory conditions [9]. *Chlorella* is a rich source of good-quality protein with amino acids, polysaccharides, lipids, vitamins, minerals, and nutrient-rich bioactive substances, presuming numerous physiological activities [10,11]. High concentrations of photosynthetic pigments and many primary carotenoids, such as *α*-carotene, *β*-carotene, lutein, ascorbic acid, and *α*-tocopherol, have been reported in *C. vulgaris*. These carotenoids, *β*-1,3 glucan, and phenolics are active immunostimulators and have the ability to scavenge free radicals and blood cholesterol. Vitamin B12 in *Chlorella* biomass is vital for blood cell formation and regeneration [12,13]. There are diverse reports on the biological activities of *Chlorella*, such as antibacterial, antiviral, antioxidant, antitumor, anti-inflammatory, and immunomodulatory activities [14,15,16,17,18].

Recent studies have emphasized the utilization of microalgae in the field of pharmaceuticals. Diverse research is now dealing with the antimicrobial activity of solvent extracts derived from microalgae [15]. The antibacterial activities of the solvent extract of *Chlorella* against fish pathogens have been reported [15,19].

Several studies have addressed that different freshwater microalgae are used as immunostimulants in fish [5,20]. A *Chlorella* incorporated diet plays a major role in immune stimulation in juvenile Korean rockfish and Koi carp [21,22]. Feeding with *Chlorella* also enhanced survival and growth rates in larval Korean rockfish and juvenile Japanese flounder [23,24]. In Gibel carp farming, an optimum level of *Chlorella* additive feed promotes the immunity, growth, and enzymatic activities of the digestive system [25]. The mass production of the ketocarotenoid astaxanthin from *C. zofingiensis* is commercially used in colorants, feed additives, and health products [26]. Crude polysaccharide extract from *C. stigmatophora* showed an immunosuppressant effect in BALB/c mice [14,27]. A recent study revealed that dietary *Spirulina*, *Chlorella*, and their mixture inclusions in Nile tilapia fish have an immunostimulatory and protective effect against bacterial infection [18,28].

The development of microalgal-based bioactive compounds for the aqua field could minimize antibiotic resistance. This study aims to explore the optimum level of *Chlorella* in a diet as an immunostimulant and the protective role of *Chlorella* against *A. hydrophila* in *L. rohita,* a candidate species contributing to two thirds of the production from carp polyculture in India.

## 2. Materials and Methods

### 2.1. Algal Collection and Preparation

*Chlorella vulgaris* was collected from the cultured medium of the algal culture unit of CIFA, Bhubaneswar. A whole mass of *Chlorella* was harvested with centrifuging (7000 rpm for 10 min). The collected pellet was shade-dried and made into a powder using a mortar and pestle. Later, it was stored in an airtight container for further study.

### 2.2. Preparation of the Algal Extract

*Chlorella* powder was soaked in ethanol (1:2 *w*/*v*) for 24 h, and the extract was made solvent-free using Rotavapor (Büchii rotary evaporator-11, Flawil, Switzerland). Eventually, the residual extracts were dissolved in ethanol to make a final concentration of 10 mg mL^−1^ [15].

### 2.3. Fractionation of Extract with Column Chromatography

The ethanolic extract of the *Chlorella* (0.5 g) was partially purified with fractionation using silica gel (SRL, 100–200 mesh size) column chromatography. The solvent system for elution was finalized with a preliminary screening of a thin-layer chromatographic (TLC) study [29]. The elution was carried out successively with the solvent system, ethyl acetate/hexane (5%, 10%, 20%, 40%, 50%, 70%), 100% ethyl acetate (EA), and 100% ethanol. According to the TLC pattern, the collected eluents were mixed into a total of nine fractions. These are CF1 (1:20 EA/Hex), CF2 (1:9 EA/Hex), CF3 (1:9 EA/Hex), CF4 (1:4 EA/Hex), CF5 (2:3 EA/Hex), CF6 (1:1 EA/Hex), CF7 (7:3 EA/Hex), CF8 (100% EA), and CF9 (100% EtOH).

### 2.4. Microorganisms

For the present antibacterial screening, four strains of *A. hydrophila* (AH1, AH2, AH3, and AH4) and two strains of *Pseudomonas putida* (PP1, PP2) were collected. The other two bacterial pathogens, *A. hydrophila* (ATCC 49040) and *Micrococcus luteus* (ATCC 49732) (DIFCO, BBL-Qualis Lab), were preserved in the Fish Health Management Division, CIFA, for challenge study and lysozyme activity, respectively [30].

### 2.5. Disc Diffusion Method

Screening of the antibacterial activity of crude ethanolic extract of *Chlorella* and the various fractions against the selected fish pathogens was completed following the disc diffusion method [31]. Nutrient agar (Hi-media) plates were prepared and swabbed with a concentration of 10^7^ CFU mL^−1^ bacterial cultures. Later, the sterile filter paper disc (6 mm in diameter, Hi-media) was impregnated with 100 µg 10 µL^−1^ concentrations of extract and was loaded on the agar plate. Streptomycin (S), tetracycline (T), and bacitracin (B) were tested as positive controls. After incubation for 24 h at 37 °C, the clear zone around the disc was measured (in mm) [32].

### 2.6. Fish and Experimental Setup

The experimental setup was designed as per the previous study [33]. A total number of 360 *L. rohita* fingerlings averaging 25 ± 2 g were acclimatized in a 400 L FRP (fibre-reinforced plastic) tank with chlorine-free tap water for 30 days. The water quality was monitored at a temperature of 28 ± 1 °C, pH 7.6, dissolved oxygen 6.0 ± 0.4 mg L^−1^, hardness 90 mg L^−1^, and total alkalinity 92 mg L^−1^. Each experimental unit was provided with aeration, and fish were fed a commercial diet twice daily at 4% of body weight.

A total of 4 experimental groups of fish were set in duplicate (C0, C1, C2, and C3) with the feeding of *Chlorella* at 0, 0.1, 0.5, and 1.0 g Kg^−1^ of the basal feed for 3 months (Table 1). An individual tank contained 45 fish. At the end of the exposure period (day 90), from each duplicate tank, 20 fish were injected with a lethal dose of *A. hydrophila* (1 × 10^5^ CFU/fish). At 30-day intervals in the pre-infection period and 10 days after the infection period, blood samples from each group of fish were evaluated for haematological, biochemical, and immunostimulant assays.

### 2.7. Blood Samples

At every 30-day interval, 30 fish from each subgroup (n = 18 for serum and n = 12 for a blood sample) were anesthetized with MS-222 (0.1 ppm) for sampling. For haematological analysis, blood was collected from the caudal vein using a disposable heparinized syringe, whereas blood sampling for serum was completed without heparin and used for the analysis of biochemical and immunological indices.

### 2.8. Non-Specific Immune Assays

#### 2.8.1. Bactericidal Activity

Following the protocol of Kajita et al. [34], serum bactericidal activity was performed. An equal ratio (1:1) of bacterial suspension (*A. hydrophila*, ATCC 49040) and serum sample was mixed and kept in an incubator for 1 h at 25 °C. Thereafter, the serum–bacterial mixture was diluted 10 times with sterile PBS (pH 7.4). Then, 100 µL of the mixture was transferred to a nutrient agar plate for culture. The viable bacterial colonies were counted and the percentage of survival was calculated after 24 h incubation at 37 °C [20].

#### 2.8.2. Superoxide Anion Production

The method of Chung and Secombes [35] with minor modifications [27] was followed for the determination of superoxide anion production using Nitro Blue tetrazolium (NBT) assay. For easy cell adhesion, a blood sample of 100 µL was placed in a 0.2% poly-L-lysine buffer (100 µL, Sigma) coated 96-well flat-bottom microtitre plate. After 2 h incubation at room temperature, it was washed with Hanks balanced salt solution (HBSS, pH 7.6). Subsequently, an equal amount of NBT solution (1 µg/mL HBSS) with *A. hydrophila* of 10^4^ cells was added. The mixed sample was allowed to incubate at 37 °C for 30 min and followed by the addition of methanol to stop the reaction. In each well, blue precipitation of formazone formed after the mixing of 120 µL of 2 M potassium hydroxide and 140 µL of dimethyl sulfoxide. The OD readings were taken at 630 nm in a multiscan spectrophotometer (Biorad, Hercules, CA, USA).

#### 2.8.3. Lysozyme Activity

Following the turbidimetry method of Parry et al. [36], serum lysozyme activity was estimated. *M. luteus* ATCC 49,732 (DIFCO, BBL-Qualis Lab) suspension was prepared using 0.05 M sodium phosphate buffer (pH 6.2) as a substrate. A serum sample of 100 µL was mixed with 2 mL of *M. luteus* suspension. After 0.5 and 4.5 min, the reading of the decreased OD value was taken at the wavelength of 530 nm in a spectrophotometer at 25 °C. The volume of the sample causes a decrease in absorbance of about 0.001/min, which is expressed as the unit of lysozyme activity.

### 2.9. Analysis of Serum Biochemical Parameters

A method adopted from Lowry et al. [37] was followed for the estimation of the total protein content in the different collected serum samples. The total albumin content was measured following Doumas et al. [38], and the globulin content was measured by subtracting the value of albumin from the total protein and the ratio of albumin: globulin.

### 2.10. Determination of Haematological Parameters

The haemoglobin level of the blood samples was estimated using the cyanomethemoglobin method of Van Kampen et al. [39] and is expressed in g%. For counting the total red blood cells and white blood cells, the haemocytometer has been used and is expressed as cells per mm^3^ [40].

### 2.11. Growth Measurements

In each treatment group, the weight gain of individual fish was measured to determine the total biomass in the tank according to the adjusted feed. Using the above data, SGR and FCR were calculated following the method of Ricker [41]. After the 90 days of the feeding trial, the body weight (%) increase per day and specific growth rate for the experimental groups were expressed as:

SGR = (ln final weight − ln initial weight)/Total experimental period × 100

The FCR value was calculated as:

FCR = Feed given (dry weight)/Weight gain (wet weight) × 100

### 2.12. Challenge of Fish

For the challenge study, the pathogenicity of *A. hydrophila* (ATCC 49040) was determined in the previous experiment [30]. At the end of the 90 days of feeding, a concentration of 1 × 10^5^ CFU/fish *A. hydrophila* was injected intraperitoneally into 20 fish per replicate. The mortality rate and clinical signs were recorded for 10 days.

### 2.13. Statistical Analysis

Data were analysed using one-way analysis of variance (ANOVA). Significant differences at *p* < 0.05 were tested for the mean values. Duncan’s multiple range test (DMRT) was used for comparing the significant differences among the treatment means [42].

## 3. Results

### 3.1. Screening of Antibacterial Activity

After column chromatography and TLC, 9 fractions were obtained, out of which 3 chromatographic fractions of ethanolic extracts resulted in improved activity against *Pseudomonas* and *A. hydrophila*. The second fraction (CF2) was found to be effective against *A. hydrophila*, AH4 (15.67 ± 0.58 mm), and *P. putida,* PP1 (13.0 ± 0 mm), at a concentration of 100 mcg showed the maximum antibacterial activity. The CF5 fraction showed the highest zone of inhibition (14.0 ± 1.73 mm) against *P. putida*, PP2 (Table 2)

### 3.2. Immunological Indices

#### 3.2.1. Superoxide Anion Production

**Figure 1:**
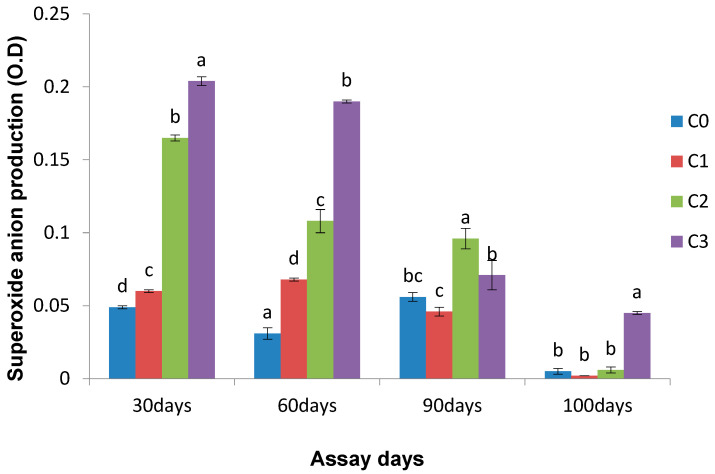
Effect of *Chlorella* on superoxide anion production on different assay days (each point represents mean ± SE). Mean values bearing different letters represent a significant difference (*p* < 0.05) between means as assessed using one-way ANOVA.

The effect of the *Chlorella* supplemented diet on the production of superoxide anion is depicted in Figure 1. On day 30 and day 60, a significant difference (*p* < 0.05) was observed in the superoxide anion values of the *C. vulgaris* supplemented diet fed fish relative to the control group. After the tenth day of bacterial challenge, a significantly higher NBT value (0.045 ± 0.001) was recorded in the serum obtained from the fish available in group C3 in comparison to the control group.

#### 3.2.2. Lysozyme Activity

**Figure 2:**
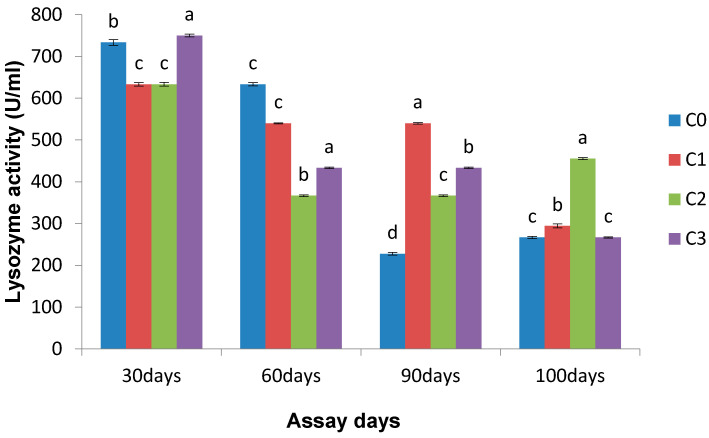
Effect of *Chlorella* on lysozyme activity (U mL^−1^) on different assay days (each point represents mean ± SE). Mean values bearing different letters represent a significant difference (*p* < 0.05) between means as assessed using one-way ANOVA.

Significantly different (*p* < 0.05) lysozyme activity was observed in the entire *Chlorella* incorporated diet group when compared with the control group on the 30th, 60th, and 90th days (except for C1 on day 60). Significantly enhanced lysozymes (750.00 ± 3.27 U mL^−1^) were recorded in the 1.0 g Kg^−1^ *Chlorella* fed fish on day 30. The entire treated group showed increased lysozyme activity after infection in comparison to the control group (Figure 2).

#### 3.2.3. Bactericidal Activity

**Figure 3:**
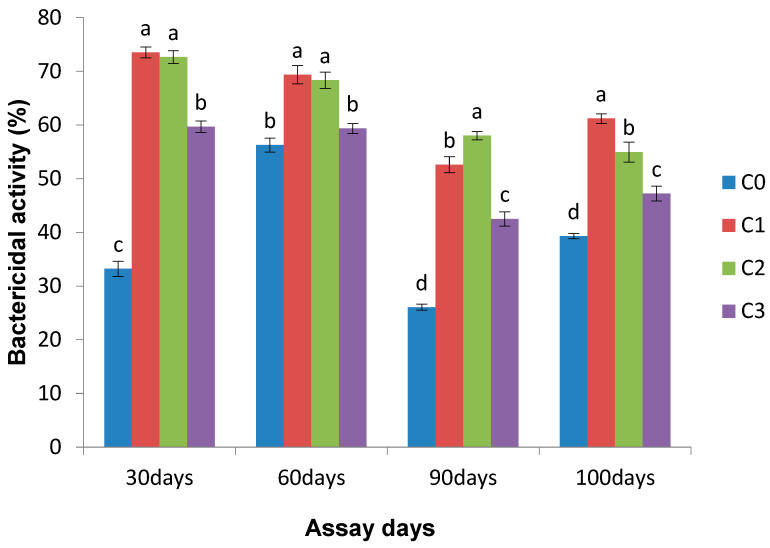
Effect of *Chlorella* on bactericidal activity (cfu control^−1^) on different assay days (each point represents mean ± SE). Mean values bearing different letters represent a significant difference (*p* < 0.05) between means as assessed with one-way ANOVA.

Elevated serum bactericidal activity was noticed in the entire experimental group when compared with the control group. On day 30, the *Chlorella* (0.1 g Kg^−1^) diet fed fish exhibited significantly (*p* < 0.05) enhanced bactericidal activity (73.52%) relative to the control diet group. A similar observation was observed in the post-infection fish (Figure 3).

### 3.3. Serum Biochemical Indices

According to Table 3, one effect of the *Chlorella* incorporated diet was significantly (*p* < 0.05) enhanced serum total protein in group C1 (0.1 g Kg^−1^ feed) on day 30 when compared with the control. After a long exposure (90 days), there was an elevated total protein level in the entire treated group relative to the control. A significantly (*p ≤* 0.05) higher post-infection total protein level was noticed in group C2 in comparison to the control. On the 30th day of the experimental period, a significantly (*p* < 0.05) different total albumin level was recorded in the fish belonging to group C1 compared to those in the control group. The fish in group C3 showed a significantly (*p* < 0.05) different albumin level on the tenth day post challenge that was lower than that of the control group as well as the other treated groups (Table 3). A significant (*p* < 0.05) difference in the total globulin value was observed in all treatment groups relative to the control group on the 90th day of the exposure period. After bacterial challenge, the treatment groups fed with 0.5 g Kg^−1^ algal powder (*C. vulgaris*) showed significantly (*p* < 0.05) increased serum immunoglobulin levels in comparison to the control group. On day 90 of the exposure, all treated groups manifested significantly (*p* < 0.05) different ratios of albumin: globulin compared to the control group. However, after bacterial challenge, a significantly (*p* < 0.05) lower albumin: globulin ratio was recorded in groups C2 and C3 when compared with the control group.

### 3.4. Effect of Chlorella on Haematological Parameters

**Figure 4:**
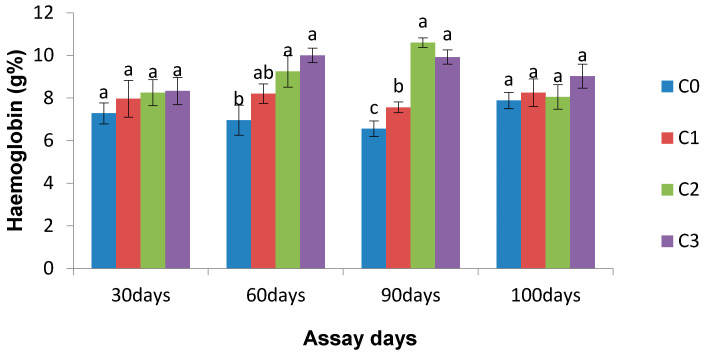
Effect of *Chlorella* on haemoglobin (g%) of *L. rohita* on different assay days (each point represents mean ± SE). Mean values bearing different letters represent a significant difference (*p* < 0.05) between means as assessed using one-way ANOVA.

*Chlorella* incorporated diet fed fishes showed an increased level of haemoglobin relative to the control group on the 30th and 10th days of the bacterial challenge, which is insignificant. However, significantly (*p* < 0.05) enhanced Hb% was noticed in all treated groups on the 60th and 90th days of the observation period (except for C1 on the 60th day) in comparison to the control group (Figure 4).

**Figure 5:**
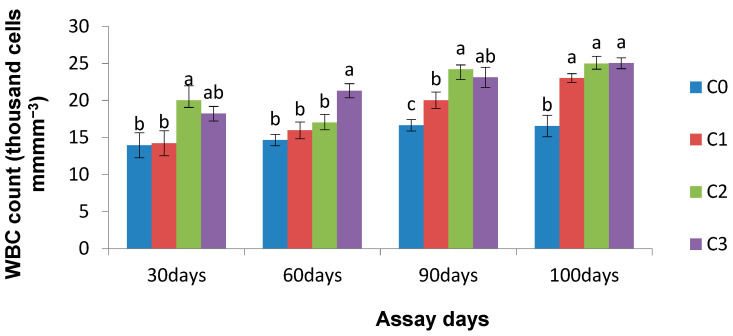
Effect of *Chlorella* on WBC count (thousand cells/mm^3^) of *L. rohita* on different assay days (each point represents mean ± SE). Mean values bearing different letters represent a significant difference (*p* < 0.05) between means as assessed using one-way ANOVA.

The WBC count was significantly higher (*p* < 0.05) in the *Chlorella* incorporated diet fed fish relative to the control group on the 90th day and 10th day of the bacterial challenge. However, an insignificant (*p* > 0.05) difference in WBC count was noticed on the 30th and 60th days of exposure (except for C2 on the 30th day and C3 on the 60th day) as compared to their respective controls (Figure 5).

**Figure 6:**
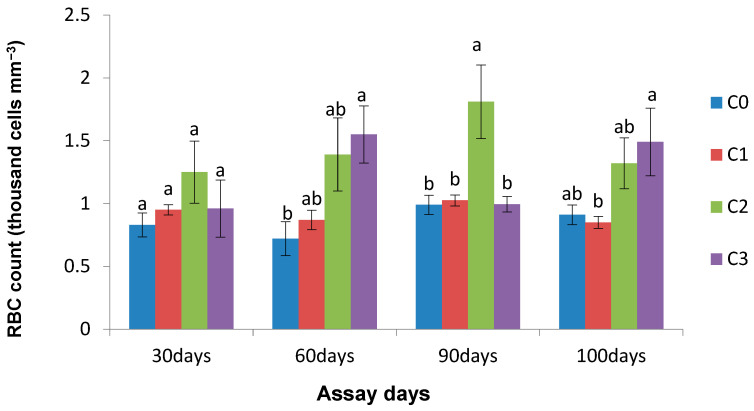
Effect of *Chlorella* on RBC count (million cells/mm^3^) of *L. rohita* on different assay days (each point represents mean ± SE). Mean values bearing different letters represent a significant difference (*p* < 0.05) between means as assessed using one-way ANOVA.

Likewise, significantly (*p* < 0.05) different total red blood cell counts were observed in groups C3 and C2 on the 60th and 90th days of the experimental period. However, an insignificantly different (*p* > 0.05) total RBC count was noticed in all treated groups in comparison to the control group on the 30th day and in the post-infection period (Figure 6).

### 3.5. Growth and Feed Efficiency

The results show that *Chlorella* was able to significantly (*p* < 0.05) increase the growth rate in the treatment groups compared to the control group. A higher specific growth rate was found in group C3 (1 g Kg^−1^ *Chlorella*). The FCR values of the fish were 1.813, 1.692, and 1.609 for the 0.1 g Kg^−1^, 0.5 g Kg^−1^, and 1 g Kg^−1^ *Chlorella* supplemented diet fed groups, respectively (Table 4).

### 3.6. Fish Survivability

Zero cumulative mortality of the fish was noticed up to 24 h in the *Chlorella* fed diet group after bacterial infection. Different dosages of *Chlorella* supplemented diet fed fish exhibited higher survival rates relative to the control group on the tenth day post infection. The maximum survival (86.5%) percentage was recorded in C3 (1.0 g *Chlorella* Kg^−1^ feed), followed by 81.65% in group C2. However, an insignificant (*p* > 0.05) difference in survivability was noticed in C2 and C3’s tanks (Table 4).

## 4. Discussion

Microalgae are rich sources of biologically active compounds and produce a large variety of secondary metabolites. The extraction, isolation, and screening of the antibacterial activity of these compounds from algae require various organic solvents [19]. From our previous investigation, it has been observed that ethanolic extracts of some microalgae show better results against pathogenic bacteria [15,31]. The present results reveal that the partially purified fractions of ethanolic extract of *Chlorella* showed high antibacterial activity against the selected bacterial pathogens (*A. hydrophila* and *P. putida*). The characterization and identification of active metabolites from microalgae that contain some pigments and fatty acids exhibiting antimicrobial activities have been conducted [15]. In this study, nine eluted fractions used a solvent system such as ethyl acetate and hexane. Further purification and characterization of these fractionated products are essential to identify the bioactive compounds with antibacterial activity.

Both the innate and adaptive immunity of fish remove foreign particles from the body. Monocytes, macrophages, granulocytes, and humoral elements are the significant constituents of the non-specific immune system [43]. The mucus and skin of fish are a unique physical barrier that acts as the first line of defence. Mostly, they contain lysozymes and immunoglobulin and complement immunoreactive molecules. The current study emphasizes the formulation of *Chlorella* incorporated fish feed. The effect of *Chlorella* powder on the immunological, biochemical, and haematological indices and growth performance of rohu fingerlings was also studied.

The current results reveal the significant immunostimulatory effect of a *C. vulgaris* supplemented diet on serum lysozyme, bactericidal activity, blood superoxide anion production (O_2_^−^), and protection against bacterial pathogens in rohu. The respiratory bursts of phagocytes produce superoxide anions and play a key role in restricting pathogenic growth [44]. Increased superoxide anion production by leucocytes has also been observed in rohu fish fed with *Euglena viridis*, *Microcystis aeruginosa*, and *Spirulina platensis* incorporated diets [20,30,45].

The enhancement of serum lysozyme activity caused by immunostimulants is due to either an increased number of phagocytes or an elevated level of synthesized lysozyme in each cell [46] The type of immunostimulants and their potency substantially influence the changes in the lysozyme activity of fish, which are exposed. Significantly enhanced serum lysozyme activity in Nile tilapia has been observed when fed with blue-green algae, *S. platensis*, at 10 mg kg^−1^ for 2–3 months [45]. The present results reveal that all treated groups on the 90th day of the trial and the 10th day post challenge exhibited increased serum lysozyme activity compared with the control group. Lysozymes in fish are bactericidal in that they rupture the peptidoglycan layer of the bacterial cell wall. A closely related result of enhanced lysozyme activity was noticed on the 30th, 60th, and 90th days after feeding Indian major carp rohu with *Euglena* [30]. Increased lysozyme activity was observed in Gibel carp with enhanced dietary *Chlorella* [25]. Increased bactericidal activity in the serum of rohu treated with *Chlorella* at various levels indicates that serum has a function of innate immunity. Many workers have revealed that herbal-immunostimulant-treated fish of different species exhibit elevated bactericidal activity [20,30,47]. The enhanced serum bactericidal activity in *Chlorella* treated groups specifies that the effective disease resistance of fish is due to the elevation of different serum humoral factors involved in non-specific immunities. Likewise, other plant-based (*M. indica* and *A*. *sativum*) and *Euglena* incorporated diet feeding enhanced the serum bactericidal activity in *L. rohita* [30,47]. The important role of macrophages is phagocytosis and destroying the invaded bacterial cell and foreign elements. The increased destruction ability of macrophages caused by cytokines and immune stimulation (beta-glucan and other compounds) induces activation of these cells [48]. It has been shown that the modulation of innate and/or adaptive immunity could be due to the involvement of *Chlorella*.

*Chlorella* may enhance the levels of total serum protein, albumin, and globulins in *L. rohita* fingerlings. Globulins play a significant role in immune systems and are synthesized in mononuclear phagocytes [49]. The long-term-fed *Chlorella* diet fish group had increased serum total protein in comparison to the control group. The present results reveal that serum albumin and globulin were also elevated in the *Chlorella* supplemented diet fed fish compared to the control group. A similar type of observation was noticed in Gibel carp fed with 2% *Chlorella* for 60 days [25]. A stronger innate immunity in fish is connected with enhanced serum protein, albumin, and globulin content [50]. Similar observations were noticed in fish fed with *Chlorella* protein (2.0 g Kg^−1^ and 4.0 g Kg^−1^) that had significantly elevated liver protein and serum albumin than fish fed with other diets [51]. The assessment of various diseases in the mammalian liver and kidney can be performed by determining the ratio of albumin and globulin. The unchanged albumin–globulin ratio after a 90-day feeding trial indicates the maintenance of the health status of the *Chlorella* treated fish.

The leukocyte plays a significant role in the defence mechanism of fish [52]. In this study, the *Chlorella* supplemented diet fed fish showed significantly increased WBC counts on day 90 and after bacterial infection. Our previous study revealed that rohu fingerlings fed with *E. viridis* showed significantly increased WBC counts [30]. Ganeshamurthy et al. [53] reported significantly increased white blood cells in clownfish when injected with purified seaweed extract. Long-term *Chlorella* fed fish showed significantly increased Hb% and total erythrocytes compared to the control group. After bacterial infection, neither the haemoglobin content nor RBC was remarkably changed.

Various microalgae have been utilized as a fishmeal replacement in aqua feed because they are a rich source of protein content. The growth performance of the rohu fingerlings observed in this study and the results reveal a higher specific growth in the *Chlorella* feeding groups containing 0.5 g Kg^−1^ and 1 g Kg^−1^ feed. Similarly, the optimum dietary *Chlorella* inclusion is approximately 0.5% and 1.2% for improved growth performance and feed utilization in juvenile Korean rockfish and Gibel carp, respectively [21,25]. A *Chlorella* incorporated diet (5%) could also improve growth in *Cyprinus carpio* [54]. Enyidi [10] observed that the SGR was best in 25% *Chlorella* incorporated diet fed catfish with the lowest food conversion ratio. So, the improved growth rate in fish might be due to the chlorella growth factor (CGF) present in *Chlorella* [9].

After bacterial infection with *A. hydrophila*, there was an increased survivability rate in the *Chlorella* incorporated diet fed fish. The cumulative survival rate was over 80% in the 0.5 g Kg^−1^ and 1.0 g Kg^−1^ *Chlorella* treated groups relative to the control group (which was over 50%). The above findings suggest that the higher protective effect against the pathogens could be due to the enhanced innate immunity in the post-challenge groups of fish.

## Figures and Tables

**Table 1:** Amount of the ingredients (for 1 kg feed) in basal and experimental diets with desired crude protein and lipid levels.

Ingredients	Group C0 (Control)	Group C1	Group C2	Group C3
Groundnut oil cake	400 g	400 g	400 g	400 g
Fish meal	250 g	250 g	250 g	250 g
Rice bran	200 g	199.9 g	199.5 g	199 g
Soyabean meal	120 g	120 g	120 g	120 g
Vitamin and mineral mixture	20 g	20 g	20 g	20 g
Starch	10 g	10 g	10 g	10 g
*Chlorella* powder	-	0.1	0.5	1.0

Calculated crude protein 400 g kg^−1^ diet. Calculated lipids 50 g kg^−1^ diet. Composition of vitamin–mineral premix (Suplevite M) used in feed formulation (in 1 kg) from Sarabhai Chemicals, Wadi, Baroda, India.

**Table 2:** Antibacterial screening of fractions against selected fish pathogens.

Fractions			*A. hydrophila*				*P. putida*	
Sl. No.	Code No.	Disc Potency (mcg)	AH1	AH2	AH3	AH4	PP1	PP2
			Zone of Inhibition (in mm)					
1	CF2	100	8.0 ± 0	12.33 ± 0.58	15.0 ± 0	15.67 ± 0.58	13.0 ± 0	12.33 ± 0.58
2	CF4	85	9.33 ± 0.58	9.67 ± 0.58	8.0 ± 0	11.0 ± 1.0	12.33 ± 0.58	12.67 ± 0.58
3	CF5	100	13.67 ± 0.58	12.0 ± 1.0	9.33 ± 0.58	11.67 ± 0.58	13.33 ± 0.58	14.0 ± 1.73
4	Clotrimazole (10 mcg)	-	11	11	-	-	10	-
5	Tetracycline (25 mcg)	-	20	29	-	-	15	-
6	Furazolodone (50 mcg)	-	23	23	-	-	15	-

**Table 3:** Showing the dietary effect of *Chlorella vulgaris* on some biochemical parameters of *L. rohita*.

Parameters	Groups	Pre Challenge			Post Challenge
		30 Days	60 Days	90 Days	10 Days
Total Protein (g dL^−1^)	C0	1.95 ± 0.05 ^b^	1.37 ± 0.17 ^b^	2.14 ± 0.20 ^c^	2.12 ± 0.52 ^b^
	C1	2.61 ± 0.21 ^a^	1.43 ± 0.16 ^b^	2.63 ± 0.11 ^bc^	2.32 ± 0.38 ^b^
	C2	1.76 ± 0.15 ^b^	2.57 ± 0.21 ^a^	2.72 ± 0.06 ^b^	4.56 ± 0.24 ^a^
	C3	1.38 ± 0.37 ^b^	1.66 ± 0.33 ^b^	3.66 ± 0.17 ^a^	2.01 ± 0.81 ^b^
					
Albumin (g dL^−1^)	C0	0.44 ± 0.01 ^a^	0.95 ± 0.02 ^a^	1.44 ± 0.10 ^a^	1.46 ± 0.13 ^a^
	C1	0.31 ± 0.01 ^b^	0.87 ± 0.02 ^a^	1.33 ± 0.31 ^a^	1.45 ± 0.09 ^a^
	C2	0.41 ± 0.02 ^a^	0.98 ± 0.05 ^a^	1.27 ± 0.35 ^a^	1.35 ± 0.09 ^a^
	C3	0.26 ± 0.02 ^b^	0.60 ± 0.01 ^b^	1.54 ± 0.22 ^a^	0.97 ± 0.01 ^b^
					
Globulin (g dL^−1^)	C0	1.55 ± 0.10 ^ab^	0.44 ± 0.03 ^b^	0.70 ± 0.01 ^b^	0.65 ± 0.12 ^b^
	C1	2.30 ± 0.21 ^a^	0.55 ± 0.19 ^b^	1.30 ± 0.07 ^a^	0.87 ± 0.07 ^b^
	C2	1.28 ± 0.51 ^ab^	1.60 ± 0.22 ^a^	1.44 ± 0.02 ^a^	2.45 ± 0.20 ^a^
	C3	1.13 ± 0.28 ^b^	1.06 ± 0.30 ^ab^	1.52 ± 0.28 ^a^	1.03 ± 0.38 ^b^
					
A: G Ratio	C0	0.27 ± 0.06 ^b^	2.09 ± 0.09 ^a^	2.66 ± 0.31 ^a^	2.14 ± 0.20 ^a^
	C1	0.13 ± 0.02 ^b^	1.79 ± 0.32 ^a^	0.99 ± 0.28 ^b^	1.69 ± 0.19 ^a^
	C2	0.45 ± 0.08 ^b^	1.24 ± 0.39 ^ab^	0.88 ± 0.29 ^b^	0.60 ± 0.23 ^b^
	C3	0.23 ± 0.01 ^ab^	0.53 ± 0.04 ^b^	0.92 ± 0.24 ^b^	0.86 ± 0.11 ^b^

Note: In each column, superscript lowercase letters a, b and c show significant difference (*p* < 0.05) compared between treatment group and control group. Resulting data are expressed as mean ± SE.

**Table 4:** Effect of oral administration of *Chlorella* on SGR and FCR, determined at the end of the experimental period of 90 days, and survivability (%) as recorded on 10th day post challenge with *A. hydrophila* on experimental fish, *L. rohita.* Mean values bearing different superscript lowercase letters a, b and c represent a significant difference (*p* < 0.05) between means as assessed using one-way ANOVA.

Group (Dose in g kg^−1^)	SGR	FCR	Survivability (%)
C0 (0)	0.86 ± 0.016 ^b^	1.83 ± 0.027 ^a^	51.65 ± 1.65 ^c^
C1 (0.1)	0.88 ± 0.014 ^b^	1.81 ± 0.024 ^a^	68.30 ± 5.00 ^b^
C2 (0.5)	0.96 ± 0.016 ^a^	1.69 ± 0.029 ^b^	81.65 ± 1.65 ^a^
C3 (1.0)	0.98 ± 0.016 ^a^	1.61 ± 0.026 ^b^	86.50 ± 3.35 ^a^

## Data Availability

The data that support the findings of this study are available on request from the corresponding author.
